# Protectors of Wellbeing During the COVID-19 Pandemic: Key Roles for Gratitude and Tragic Optimism in a UK-Based Cohort

**DOI:** 10.3389/fpsyg.2021.647951

**Published:** 2021-07-08

**Authors:** Jessica P. Mead, Zoe Fisher, Jeremy J. Tree, Paul T. P. Wong, Andrew H. Kemp

**Affiliations:** ^1^Department of Psychology, Swansea University, Swansea, United Kingdom; ^2^Fieldbay, Swansea, United Kingdom; ^3^Traumatic Brain Injury Service, Morriston Hospital, Swansea, United Kingdom; ^4^Health and Wellbeing Academy, Swansea University, Swansea, United Kingdom; ^5^Department of Psychology, Trent University, Peterborough, ON, Canada

**Keywords:** wellbeing, positive psychology, gratitude, tragic optimism, COVID-19, second wave positive psychology, GENIAL model

## Abstract

The COVID-19 pandemic has presented a global threat to physical and mental health worldwide. Research has highlighted adverse impacts of COVID-19 on wellbeing but has yet to offer insights as to how wellbeing may be protected. Inspired by developments in wellbeing science and guided by our own theoretical framework (the GENIAL model), we examined the role of various potentially protective factors in a sample of 138 participants from the United Kingdom. Protective factors included physical activity (i.e., a health behaviour that helps to build psychological wellbeing), tragic optimism (optimism in the face of tragedy), gratitude (a prosocial emotion), social support (the perception or experience of being loved, cared for, and valued by others), and nature connectedness (physical and psychological connection to nature). Initial analysis involved the application of one-sample *t*-tests, which confirmed that wellbeing (measured by the Warwick-Edinburgh Mental Well-being scale) in the current sample (*N* = 138; *M* = 46.08, SD = 9.22) was significantly lower compared to previous samples (*d* = −0.36 and *d* = −0.41). Protective factors were observed to account for up to 50% of variance in wellbeing in a hierarchical linear regression that controlled for a range of sociostructural factors including age, gender, and subjective social status, which impact on wellbeing but lie beyond individual control. Gratitude and tragic optimism emerged as significant contributors to the model. Our results identify key psychological attributes that may be harnessed through various positive psychology strategies to mitigate the adverse impacts of hardship and suffering, consistent with an existential positive psychology of suffering.

## Introduction

COVID-19 is a respiratory virus leading to general symptoms such as fever and cough, with more severe cases requiring intubation (Chan et al., 2020; Wang et al., 2020). On March 11th 2020, the World Health Organisation (WHO) declared the COVID-19 outbreak a global pandemic and on March 23rd, the UK government declared a nation-wide lockdown requiring citizens to stay at home. Residents were only permitted to leave their household to shop for basic necessities, to exercise once a day, to tend to medical needs, or to travel for work when working from home was not possible. Since the easing of the first nation-wide lockdown further restrictions had been imposed in the United Kingdom although these differed by locality. As of February 22nd 2021, over 109 million cases had been diagnosed globally with more than 2.3 million fatalities (GOV.UK, 2020). Beyond threat to life, COVID-19 has caused widespread bereavement, self-isolation, loss of income, unemployment as well as delays in treatment for ongoing health conditions as resources are diverted toward managing COVID-19 patients (Spinelli and Pellino, 2020).

Recent publications on the COVID-19 pandemic have raised concerns about the deterioration of mental health (Cullen et al., 2020; Galea et al., 2020; Gunnell et al., 2020; Pfefferbaum and North, 2020; Xiong et al., 2020). The Office for National Statistics (ONS, 2020) reported a large increase in anxiety and decrease in life satisfaction due to boredom, loneliness, anxiety, and stress during March and April, 2020 (ONS, 2020), representing the first 2 months of lockdown associated with the COVID-19 pandemic. A significant reduction in wellbeing—measured by the Warwick-Edinburgh Well-being Scale—was observed in a large sample of 12,989 participants from Wales (Gray et al., 2020). Other researchers have highlighted potentially protective factors against loneliness, including higher levels of social support (*OR*: 0.92), being married or co-habiting (*OR*: 0.35), and living with more adults (*OR*: 0.87) (Groarke et al., 2020). Higher levels of community connectedness have also been linked to lower levels of psychological distress (Sibley et al., 2020). While this research has improved our understanding of the factors that can protect against ill-being during the pandemic, reducing ill-being is not the same as promoting wellbeing, as wellbeing does not necessarily emerge when illbeing is reduced (Ryff et al., 2006; Westerhof and Keyes, 2010). Furthermore, our own work has shown that wellbeing is possible despite much suffering (Fisher et al., 2020; Tulip et al., 2020; Wilkie et al., 2021). Theoretical developments now emphasise that navigating the challenges of life and experiencing suffering may actually contribute to sustainable wellbeing (Wong, 2020a) and post-traumatic growth (Chan et al., 2016). In addition, researchers have argued for the use of self-guided therapeutic and positive psychological approaches to manage wellbeing during self-isolation and social distancing, including physical activity, savouring positive emotions, and optimising positive social resources (Fischer et al., 2020a; Holmes et al., 2020; Yamaguchi et al., 2020). It is therefore important to understand the extent to which positive psychological factors contribute toward wellbeing during a time of great individual and societal suffering. Accordingly, the aim of our study is to better understand the factors that may help to protect and build wellbeing during the COVID-19 pandemic.

Our focus on wellbeing during the pandemic is considered within the context of our GENIAL model—*Genomics—Environment—vagus Nerve—social Interaction—Allostatic regulation—Longevity—*(Kemp et al., 2017; Mead et al., 2019, 2021; Fisher et al., 2020), a theoretical framework of wellbeing, which we have defined as “positive psychological experience,” underpinned by connection to ourselves, to others and to the environment within which we live (Mead et al., 2019, 2021). The GENIAL model is a life-course biopsychosocial framework that places individual wellbeing within the context of their social and natural ecologies. The framework encourages reflection on how wellbeing might be improved by targeting features across individual (e.g., positive emotions, and physical health behaviours), community (e.g., social support and connection), and environmental domains (nature connectedness). While these domains broadly reflect higher levels of scale consistent with Bronfenbrenner's adapted multi-levelled ecological systems theory (Lomas, 2015), there is scope for the individual to build wellbeing within each of these domains. For the present study, we chose variables—shown in previous work to contribute to wellbeing—from each of these domains. There is also scope for higher levels of scale to impact on wellbeing within each of these domains beyond the immediate control of the individual including age, gender, and socioeconomic status (World Health Organisation Calouste Gulbenkian Foundation, 2014). We now briefly review the evidence linking each of these chosen exemplars to wellbeing, providing the rationale for our focus on these factors.

The individual domain of our theoretical framework emphasises a role for positive attributes such as optimism and engagement in physical activity, drawing on published evidence demonstrating the impacts of mind and body interventions on wellbeing. In regards to “mind,” we focus here on the role of tragic optimism (Wong, 2019a) in particular, which is a construct defined as “optimism in the face of tragedy” and in spite of pain, guilt, and death (the “tragic triad”). Tragic optimism differs from more traditional optimism as it places an emphasis on hope despite distress and suffering, and therefore has relevance to the experience of living through the ongoing pandemic of COVID-19. Research has shown that daily optimism during the pandemic is positively associated with support from others (Kleiman et al., 2020), demonstrating a link between the positive psychological attribute of optimism and one's capacity for connecting to others. Studies have also reported associations between optimism and multiple health factors, ranging from small to large effects, including quality of life (*r* = 0.37), mental health (*r* = 0.21; Auer et al., 2016), and subjective wellbeing (*r* = 0.54; Duy and Yildiz, 2019). A meta-analysis further demonstrated a relationship between optimism and coping (Nes and Segerstrom, 2006), such that optimism is associated with coping strategies to manage stress or emotion (*r* = 0.17).

In addition to tragic optimism, we also focus on the life orientation of gratitude in which one displays an appreciation generally (McCullough et al., 2002) is benefical for wellbeing. To highlight this point, a recently published meta-analysis of 158 independent samples on more than 100,000 participants concluded that dispositional gratitude is moderately to strongly correlated with well-being (Portocarrero et al., 2020). Importantly, recent work highlighted that higher levels of gratitude early in the pandemic (January – March) predicted lower psychological harm (*B* = −0.239) and higher subjective wellbeing (*B* = 0.584) among a small sample (*N* = 86) a few months later (April–May) (Bono et al., 2020). Individuals with a grateful disposition are more likely to appreciate other people (McCullough et al., 2001; Gulliford et al., 2013; Ma et al., 2017), highlighting a role for gratitude in prosocial behaviour (Ma et al., 2017). In addition, a grateful disposition leads people to appreciate life in general (Wood et al., 2010). Despite different theoretical approaches to gratitude (and their respective measures), all support a higher order gratitude factor relating to a life orientation of gratitude (Wood et al., 2010). As with an optimistic life orientation, one with a grateful life orientation would experience a greater frequency and intensity of gratitude regardless of measure used, as argued by Wood et al. (2008b). Wood et al. (2010) highlights that gratitude has been associated with a variety of adaptive personality traits, multiple conceptions of wellbeing, post-traumatic growth and is inversely associated with poor health behaviours and poor mental health.

Further to positive psychological attributes, recent meta-analyses have highlighted a role for positive health behaviours, such as physical activity, for improving wellbeing. For instance, a recent meta-analysis on 157 studies reported a beneficial small effect of physical activity on subjective wellbeing *d* = 0.36, 95% CI [0.301, 0.420] (Buecker et al., 2020). Given the extensive barriers to exercise during the pandemic (due to closure of indoor public spaces and restrictions on the number of times allowed to leave the house), researchers have argued that increasing physical activity levels should be prioritised as a treatment target in psychological therapy (Diamond and Waite, 2020). Further research has supported the benefits of physical activity during the pandemic for wellbeing in the UK, with fewer hours of moderate-to-vigorous physical activity per day associated with poorer mental wellbeing (as measured by the short Warwick-Edinburgh Mental Wellbeing Scale), *OR* = 0.82, 95% CI [0.67–0.98] (Jacob et al., 2020). Physical activity has also been associated with increased levels of psychological wellbeing during the pandemic in Italy (Maugeri et al., 2020), and is the most commonly reported coping behaviour during the pandemic among healthcare workers in New York City (Shechter et al., 2020).

Our GENIAL model (Kemp et al., 2017; Mead et al., 2019, 2021) further emphasises a role for community and social ties as a major determinant of health and wellbeing, a topic that has been labelled “the new psychology of health” (Haslam et al., 2017). A major protective factor within the community domain is social support, defined as the perception or experience of being loved, cared for, and valued by others. Social support has been shown to be positively related to wellbeing measures, such as life satisfaction (*r* = 0.23) and personal wellbeing (*r* = 0.34) (Brajša-Žganec et al., 2018). A highly cited meta-analysis of 148 studies reported a 50% increased likelihood of survival for participants with stronger social relationships (indicated by social support; *OR* = 1.50, 95% CI [1.42–1.59]) (Holt-Lunstad et al., 2010). There is an extensive list of mechanisms through which social support may positively impact on health and wellbeing, including behavioural (e.g., health behaviours), psychological (e.g., quality of life) pathways, and biological pathways (e.g., immune function) (Thoits, 2011; Uchino et al., 2018). As noted earlier, social support is playing a vital role in reducing illbeing during the COVID-19 pandemic (Groarke et al., 2020; Sibley et al., 2020). Coping strategies involving social support have proven beneficial for wellbeing during the pandemic for those living in Germany, with emotional support being associated with increased positive affect (*B* = 0.11) and instrumental support (in the form of advice) being associated with increased life satisfaction (*B* = 0.06) (Zacher and Rudolph, 2021).

Recent iterations of our GENIAL model (Mead et al., 2019, 2021) further highlights a contributing role for the natural environment to wellbeing, an especially important consideration in light of the observed and predicted impacts of anthropogenic climate change (Cook et al., 2016) and potential ecosystem collapse (Future Earth, 2020). Recent work on a representative sample of the adult population from England demonstrated that a physical and psychological connection to nature, known as “nature connectedness,” contributes to wellbeing and may even play a role in promoting pro-environmental behaviour (Martin et al., 2020). The relationship between nature connection and eudaimonic wellbeing (*r* = 0.24) as well as hedonic wellbeing (*r* = 0.20) (Pritchard et al., 2020) is associated with small to medium effect sizes. It has even been argued that connecting people to nature could provide a population-wide strategy for health promotion (Maller et al., 2006) that may help to tackle health inequities (Allen and Balfour, 2014) while contributing to pro-environmental behaviours (Richardson et al., 2020). Interestingly, research during the pandemic in Canada highlighted that among both active and inactive individuals, those classified as flourishing indicated greater nature relatedness compared to those who scored low on the scale (Lesser and Nienhuis, 2020), therefore indicating that nature connection plays an important role for wellbeing regardless of physical activity levels. Research across 9 countries (*N* = 5,218) highlighted that people believed a view of nature and contact with nature helped buffer the negative effects of lockdown and increased positive emotion (Pouso et al., 2020). The researchers argued that ecosystems provide additional opportunities to mitigate the negative impacts of pandemic-related lockdowns. However, despite research highlighting the benefit of green spaces on wellbeing, research has highlighted a reduction in the use of urban green spaces by respondents in many European countries during the pandemic, compared to pre-pandemic use, possibly due to the lockdown restrictions (Ugolini et al., 2020).

While the above factors are discussed as independent contributors to wellbeing, they are all interrelated and inter-connected components of a wider framework (GENIAL) that may promote each other to some degree (Elavsky et al., 2005; Chen and Kee, 2008; Holt-Lunstad et al., 2010; Kok et al., 2013; Dadvand et al., 2016; Petersen et al., 2019). We refer the reader to our GENIAL model for further information on the inter-relationships between the factors (Kemp et al., 2017; Mead et al., 2019, 2021; Fisher et al., 2020). The nation-wide, lockdown associated with the COVID-19 pandemic in the UK provided a unique opportunity to explore the impact on and contributors to wellbeing during a time of great suffering, the focus of Second Wave Positive Psychology (PP 2.0), also described as existential positive psychology (Wong, 2020b; Wong et al., 2020). Our study sought to test several predictions. First, it was predicted that wellbeing would be significantly lower than that reported in surveys on UK samples prior to COVID-19—consistent with recent research reporting the same (Gray et al., 2020)—providing a platform on which results from additional analysis would be interpreted. Second, we predicted that physical activity, gratitude, tragic optimism, social support, and nature connection would act to protect wellbeing during the pandemic, over and above the impacts of sociostructural factors including age, gender, and subjective social status.

## Method

### Participants

A total of 138 UK residents participated voluntarily in this study, including 109 females and 29 males, with a mean age of 33.32 (SD = 13.32), ranging from 18- to 68-years. Participants were recruited via advertisements on social media platforms and an internal departmental advertisement site. The research protocol was considered and approved by the Department of Psychology ethics committee at Swansea University (approval number: 2020-3862-2832).

### Measures

At the time this study was carried out, it was not clear how long the lockdown would remain in place. Limitations were therefore imposed on the length of chosen measures to ensure that the time taken to complete the survey maximised potential recruitment and minimised potential attrition.

### Physical Activity

A single item was used to measure physical activity in which participants were asked how physically active they had been on a 5-point Likert-type scale from a value of 1 (not at all active) to 5 (extremely active) during the previous 2 weeks. A single item to measure physical activity has several advantages including brevity and parsimony, and has been shown to be both reliable and valid (Schechtman et al., 1991; Milton et al., 2011; Gill et al., 2012; Portegijs et al., 2017; O'Halloran et al., 2020).

### Gratitude

The Gratitude Questionnaire-Six-Item Form (GQ-6) (McCullough et al., 2002) is a six-item questionnaire based on a Likert scale from 1 (strongly disagree) to 7 (strongly agree). Items 3 and 6 are reversed scored, after which all scores are then added to obtain a total score out of 42. The GQ-6 has relatively high internal consistency (Cronbach's alpha ranging from 0.76 to 0.87), convergent validity *(r* = 0.33, *p* < 0.01; McCullough et al., 2002) and temporal validity (*r* = 0.59 and 0.73 for two samples; Wood et al., 2008b). Discriminant validity was indicated by factorial independence of the GQ-6 from measures of related constructs, these being life satisfaction (*r* = 0.53), vitality (*r* = 0.46), happiness (*r* = 0.50), tragic optimism (*r* = 0.51), and hope (*r* = 0.67; McCullough et al., 2002).

### Tragic Optimism

The Life Acceptance Measure (LAM; Wong, 2019a) is a new 9-item measure with statements on a 5-point Likert scale (1 being strongly disagree and 5 being strongly agree), with a Cronbach's alpha score of *a* = 0.82 (see Supplementary Material). The scores are added, and a total is obtained. The maximum score is a total of 45.

### Social Support

The Multidimensional Scale of Perceived Social Support (MSPSS) is a 12-item scale designed to measure perceived social support from family, friends, and a “special person” (Zimet et al., 1988). The measure uses a 7-point Likert scale, ranging from 1 (very strongly disagree) to 7 (very strongly agree). Scores are added and a total is obtained with a maximum score of 84. The scale has good internal reliability, with Cronbach's alpha ranging from 0.84 to 0.92, and has moderate to strong factorial validity and construct validity (Zimet et al., 1988, 1990).

### Nature Connection

Previous questionnaires have focused on either contact with (Largo-Wight et al., 2011) or connection to nature (Mayer and Frantz, 2004; Nisbet and Zelenski, 2013). We argue that both are important for wellbeing, but inclusion of multiple existing measures would lengthen our survey unnecessarily. Accordingly, and for brevity, a new measure named “Nature Connection” was created, to measure physical as well as psychological connection to nature. The statements are (1) “I feel I spend enough time in nature,” (2) “I wish I could spend more time in nature,” (3) “I feel disconnected from nature,” and (4) “I am often immersed in nature.” Responses ranged from 1 (strongly disagree) to 5 (strongly agree). Respondents were informed that the term nature referred to green spaces (such as parks, forests, gardens, fields) and blue spaces (such as lakes, rivers, the sea) and were asked to respond based on their experiences during the past 2 weeks. Cronbach's alpha indicated that statement 2 needed removing (as this statement was reducing the reliability), leading to a three-item measure relating to nature connectedness. Following removal of this item, Cronbach's alpha increased from 0.719 to 0.777 (see Supplementary Material). A summary measure is calculated by reverse scoring item 3, after which all items are added together, providing a total score out of 15.

### Wellbeing

The Warwick-Edinburgh Mental Well-being Scale (WEMWBS) is a positively worded 14-item measure on a 5-point Likert scale (1-5) that measures subjective and psychological wellbeing (Tennant et al., 2007). Prior research has indicated a Cronbach's alpha score of 0.89 (student sample) and 0.91 (population sample) and correlations with other measures of mental health and wellbeing indicate convergent validity (Tennant et al., 2007). Authors also noted that test-retest reliability was 0.83, 1 week between assessments. Item scores were added to produce a total score. The maximum score is a total of 70. Data collected for this study was compared with data reported in the 2018 Scottish Health Survey (*N* = 4,810 adults) (Cheong et al., 2018), and the Health Survey for England 2016 (*N* = 8,011; Morris and Earl, 2017).

### Covariates

Covariates included socioeconomic status (SES), age, and gender, all of which influence wellbeing (World Health Organisation Calouste Gulbenkian Foundation, 2014). The MacArthur Scale of Subjective Social Status (SSS) is a measure of subjective social status relating to socioeconomic position (Adler et al., 2000) with greater sensitivity for assessing SES, compared to questions on income and/or education level. The MacArthur Scale of SSS has previously predicted health and wellbeing better than objective measures of SES (Singh-Manoux et al., 2005).

### Design and Procedure

Using a cross-sectional design, data collection commenced on 8th April 2020, 16 days after lockdown was introduced in the UK, and ceased on 23rd May 2020, lasting 45 days. Participants accessed an anonymous online link to the questionnaire, hosted on the Qualtrics platform. Participants were informed of questionnaire content and consent was provided via a tick box, prior to questionnaire completion. The first part of the questionnaire focused on demographic items and subjective physical activity, after which respondents were presented with remaining measures in random order, asking them to reflect on their experiences during the preceding 2-week period.

### Statistical Analysis Method

Of the exported data from Qualtrics (*N* = 220), those who did not proceed beyond the information sheet (*N* = 13), who did not provide age (*N* = 25), who provided age but were under 18 years old (*N* = 3), who were not from the UK (*N* = 28), who did not provide SES (*N* = 3), and who had at least one value missing from the wellbeing measure (*N* = 9) were removed. In addition, one participant was flagged for completing the questionnaire in a short period of time (304 s). Upon inspection, they were suspected of satisficing (more specifically, straight-lining), and were therefore removed. This resulted in 138 participants for the demographic information and one sample *t*-test. Further participants were removed for the regression if they had at least one missing value in any of the measures included in the analysis (*N* = 15), resulting in 123 participants.

Statistical tests were conducted using SPSS and JASP. One-sample *t*-tests were carried out to compare the wellbeing data with previous UK-based samples. For the regression, SES and physical activity were converted into dummy variables. For SES, “low” was determined as a score of 0–4, “middle” was determined as a score of 5 or 6, and “high” was determined as a score of 7–10. For physical activity, a score of 1 or 2 was classed as “low,” 3 was classed as “moderate,” and 4 or 5 was classed as “high.” The reference variable for SES and physical activity was “low SES” and “low physical activity,” respectively. A two-step, hierarchal, linear regression was conducted using the enter method to determine whether predictor variables significantly protected wellbeing during the lockdown, while controlling for age, gender, and subjective SES. The first step of the model included age, gender, and subjective SES, as those variables are key influencers of wellbeing lying beyond the individual control. The protective factors were collectively added in the second step, consistent with the GENIAL model, which characterises three overlapping and interacting domains to protect wellbeing (including individual, community, and environment domains). Effect sizes (*d* and *r*) and Bayes factors are reported to illustrate the size of the effect and degree of support for the null and alternative hypothesis. Effect sizes are described as either small (*d* = 0.2, *r* = 0.1), medium (*d* = 0.5, *r* = 0.3), or large (*d* = 0.8, *r* = 0.5) based on benchmarks suggested by Cohen (1988). Bayes factors were determined using the Summary Statistics module in JASP version 0.13.1 (Ly et al., 2018). A classification scheme for interpreting Bayes Factors (Jeffreys, 1961; Lee and Wagenmakers, 2013; Wagenmakers et al., 2018) is used such that values of 1 to 3 correspond with anecdotal evidence, values of 10 to 30 as strong evidence, values of 30 to 100 as very strong evidence, while values exceeding 100 reflect extreme evidence in support of the null (BF_01_) or alternative (BF_10_) hypothesis.

## Results

### Descriptive Statistics

The characteristics of the sample (*N* = 138) are presented in Table 1.

**Table 1:** Characteristics of sample.

**Characteristics**	**Category**	***N***	
Gender	Female	109	
	Male	29	
Age	18–27	64	
	28–37	30	
	38–47	14	
	48–57	21	
	58–68	9	
Subjective social status	0–4	25	
	5–6	53	
	7–10	60	
The presence of a physical health condition	Yes	26	
	No	110	
	Did not answer	2	
The presence of a mental health condition	Yes	22	
	No	114	
	Did not answer	2	
The presence of COVID-19 symptoms	Yes	8	
	No	130	
Physical Health	Poor	6	
	Fair	30	
	Good	46	
	Very Good	41	
	Excellent	15	
Mental Health	Poor	13	
	Fair	42	
	Good	49	
	Very Good	25	
	Excellent	9	

### Comparison of Current Sample to a Sample From the UK

A one-sample *t*-test was performed, comparing data from 138 participants with that from a Scottish general population sample from 2018 (*N* = 4,810 adults) (Cheong et al., 2018). Results highlighted a significant difference in wellbeing between the current (*M* = 46.08, SD = 9.08) and previously published sample [(*M* = 49.4, SD = 8.96), *t*_(137)_ = −4.23, *p* = 0.000, BF_10_ = 362.64] representing a small to medium effect size (*d* = 0.36) (Cohen, 1988), The average wellbeing score of the current sample was 3.32 points less than the general population sample from 2018. Comparing our sample with another from the 2016 study from England (*M* = 49.9) (Morris and Earl, 2017), results again indicated a significant reduction in our current sample [*t*_(137)_ = −4.87, *p* = 0.000; *d* = 0.41, BF_10_ = 4295.42].

### Predicting Wellbeing

A hierarchical, linear regression was performed using data from 123 participants. The assumption of linearity was met, and multicollinearity was not a concern. The outcome variable was normally distributed, and inspection of the residuals highlighted that the data was homoscedastic. In addition, the data contained no outliers and the assumption of independent errors and non-zero variances was met. See Supplementary Material for more information.

With all assumptions met, a two-step, multiple, hierarchical, linear regression was conducted to see if physical activity, gratitude, tragic optimism, social support, and nature connection predicted wellbeing, after controlling for age, gender, and SES. The descriptive statistics and correlations are provided in Tables 2, 3 below.

**Table 2:** Mean and Standard Deviation of variables.

**Measure**	**Mean**	**Standard deviation**
Wellbeing	45.83	8.84
Physical activity	3.10	1.04
Gratitude	33.38	6.43
Tragic optimism	34.00	5.26
Social support	64.82	14.40
Nature connection	9.86	3.08

**Table 3:** Zero-order correlations amongst wellbeing variables.

	**Wellbeing**	**Physical activity**	**Gratitude**	**Tragic optimism**	**Social support**	**Nature connection**
Wellbeing	1.00	0.31^**^	0.63^**^	0.54^**^	0.46^**^	0.35^**^
Physical activity	0.31^**^	1.00	0.30^**^	0.13	0.23^**^	0.39^**^
Gratitude	0.63^**^	0.30^**^	1.00	0.52^**^	0.45^**^	0.26^**^
Tragic optimism	0.54^**^	0.13	0.52^**^	1.00	0.39^**^	0.33^**^
Social support	0.46^**^	0.23^**^	0.45^**^	0.39^**^	1.00	0.18^*^
Nature connection	0.35^**^	0.39^**^	0.26^**^	0.33^**^	0.18^*^	1.00

* *p < 0.05*.

** *p < 0.01*.

Results from the first block, which contained the control variables only (age, sex, SES) were significant, *F*_(4, 118)_ = 2.62, *p* = 0.038, *R*^2^ = 0.08, *R*^2^ Adjusted = 0.05. However, SES was the only variable to significantly contribute toward this model. The addition of the predictor variables (block 2) significantly improved the model, F change (6, 112) = 18.35, *p* < 0.000, *R*^2^ Change = 0.46, *R*^2^ = 0.54, *R*^2^ Adjusted = 0.5, BF_10_ = 3.041e+12. Results from block 2 of the regression can be found in Table 4. Inspection of the Bayes Factor revealed extreme evidence for the full model relative to that with only control variables. Gratitude and tragic optimism were the only variables to contribute significantly to the model. No other predictor and control variables contributed significantly to the model. The results from the *t*-tests are presented below. Inspection of the standardised beta values highlighted that gratitude was the most influential variable in the model.

**Table 4:** Results from the regression.

	***t***	***p*-value**	**Standardised Beta value**
Gratitude	4.55	0.000	0.38
Tragic optimism	2.73	0.007	0.22
Social support	1.88	0.063	0.14
Nature connection	1.43	0.155	0.11
Physical activity (moderate)	0.86	0.393	0.07
Physical activity (high)	1.15	0.252	0.10

## Discussion

The aim of the present study was to examine the contributions of selected protective factors to a reliable and valid measure of wellbeing during the COVID-19 pandemic. We also sought to determine whether wellbeing of participants during the COVID-19 lockdown was less than that reported by other studies from the United Kingdom prior to the emergence of COVID-19 to help contextualise reported findings. As expected, we reported a significant reduction in wellbeing in our UK-based sample compared with prior samples, findings associated with a small to medium effect size. This is consistent with other research showing reductions in wellbeing in larger samples during the pandemic (Gray et al., 2020). We further observed that the protective factors accounted for up to 50% of the variance in wellbeing, in a full regression model, an especially strong finding in psychological science. Key roles of tragic optimism and gratitude emerged as significant predictors of wellbeing during a time of great suffering, core characteristics of an existential positive psychology (PP2.0) (Wong, 2011, 2019b; Wong et al., 2020).

We show here that both gratitude and optimism significantly contribute to wellbeing over and above sociostructural factors of age, sex and subjective social status and other protective factors that were included in the model. Gratitude and optimism were identified as key positive psychological attributes contributing to wellbeing. These factors reflect a “life orientation” in which one displays general appreciation and expects future outcomes to be positive (Wood et al., 2010; Carver and Scheier, 2014), respectively. Data from meta-analyses and epidemiology provide insights as to the extent of the positive impacts of gratitude and optimism. As highlighted previously, gratitude correlates with various types of wellbeing (Portocarrero et al., 2020), including emotional (such as quality of life, life satisfaction, and flourishing) and social (such as positive relationships and prosocial behaviour) wellbeing (Jans-Beken et al., 2020). The positive impact of gratitude also likely contributes to longevity, not only through different types of wellbeing, but also by reducing psychopathology (Jans-Beken et al., 2020) and improving cardiovascular health (Cousin et al., 2020), among other potential pathways. Regarding optimism, a study on two epidemiologic cohorts of people reported a dose-dependent association of higher optimism levels at baseline with increased longevity (Lee et al., 2019). Specifically, those with the highest versus lowest optimism levels had 1.5 (women) and 1.7 (men) greater odds of surviving to the age of 85 years, after adjusting for demographic and health conditions findings associated with what was described as “exceptional longevity.” Research has also highlighted that these factors can protect wellbeing during extremely distressing experiences. For example, optimism can mitigate the influence of negative and traumatic life events on suicide ideation (Hirsch et al., 2009). In the field of second wave positive psychology, tragic optimism and existential gratitude are critical components of a positive psychology of suffering (Wong, 2019b) and are essential for aiding survival and growth during adversity and trauma (Wong, 2020a). Tragic optimism may provide a conceptual roadmap for clinicians to help trauma survivors accept their traumatic experiences, and affirm meaningful and virtuous aspects of their lives (Leung, 2019). As such, it has been argued that tragic optimism and existential gratitude are needed during COVID-19 and post-pandemic world (Uppal, 2020; Wong, 2020a).

We therefore advocate for the adoption of strategies to promote the experience of gratitude and tragic optimism, through, for example, the “three good things” activity (Lai, 2017) and finding meaning from adverse experiences in order to cultivate a tragically optimistic outlook (Leung, 2019). Gratitude and optimism can enhance connectedness to oneself, others and the natural environment (Brissette et al., 2002; Bono and Sender, 2018). For example, research has highlighted that gratitude directly fosters perceived social support (Wood et al., 2008a) and may even enhance the positive impact of social support on psychological wellbeing (Deichert et al., 2019). Social support may also be a key route through which the health benefits of optimism may arise (Scheier and Carver, 1987; Brissette et al., 2002). The emotion of gratitude has even been considered to play a role in connecting individuals to the natural environment (Petersen et al., 2019). It is possible therefore that the lack of significant contribution to the regression model by protective factors other than gratitude and optimism is attributable to the inter-relationships between measured variables in the context of lockdown.

Interestingly, physical activity, social support and nature connection contributed to the regression model in terms of variation in wellbeing (evident by zero-order correlations and beta values), however, they did not independently contribute to the model over above the contributions of gratitude and tragic optimism. Further work is needed to explore potential inter-relationships among potentially protective factors, guided by new theoretical frameworks such as the GENIAL model that seek to broaden understanding of the complex construct of wellbeing by expanding focus beyond the individual to issues relating to community, the natural environment and other sociostructural factors, consistent with a systems informed positive psychology (Kern et al., 2019). Some initial work in this area has demonstrated that social support and physical activity partly mediate the relationship between nature exposure and health (Dadvand et al., 2016). Another study conducted during the pandemic—in Bulgaria—reported that the positive mental health effects of outdoor green space were partially mediated by social support (Dzhambov et al., 2020). It is possible therefore that nature may have provided a context within which social support and physical activity was experienced during lockdown.

Several limitations of the present study are worth noting. The first limitation concerns the context within which the research was conducted, by which we refer to the regulations and restrictions associated with UK lockdown. It remains to be determined as to whether results are replicable in countries where lockdown was either more restrictive or relaxed. A second limitation is the small sample size, which imposed restrictions on the number of variables able to be entered into the model. We have however included a broad range of protective factors that have been previously shown to play a contributing role to wellbeing, and after examining all of these in our regression model, gratitude and optimism emerged as key contributors to wellbeing during the pandemic. We suggest therefore that these findings provide some evidence of the importance of these factors relative to the others that were included in the regression model. A related limitation was restricting the number of measures within each of the broad domains. Ideally, we would have measured additional factors known to influence of wellbeing guided by theory (e.g., diet, sleep, meaning and purpose, social capital, cohesion, active hope and sustainable behaviour) across each of the three domains to highlight the importance of a greater variety of factors that are vital for protecting wellbeing. However, at present, there is no measure that encompasses all these variables, and it was not feasible, nor practical to administer multiple additional measures of extra variables. Instead, we chose exemplars from across the core domains guided by findings from influential meta-analyses (Nes and Segerstrom, 2006; Holt-Lunstad et al., 2010; Davis et al., 2015; Wiese et al., 2018; Pritchard et al., 2020). Our study also comprised of a relatively larger number of women (*n* = 109) than men (*n* = 29). While gender was a control variable in our study, further research on a larger sample with more equal proportion of males and females would be able to determine the extent to which the findings reported here are replicable and generalisable. Another limitation is that of self-reported, cross-sectional data, which ran the risk of desirability bias (Graeff, 2005). However, other research suggests that desirability bias does not play a significant role in self-reported wellbeing measures (Caputo, 2017). Regarding the cross-sectional nature of the study, we are not able to draw conclusions relating to causal direction. However, based on the literature of the GENIAL model (Kemp et al., 2017; Mead et al., 2019, 2021) we suggest that gratitude and tragic optimism may contribute to wellbeing, rather wellbeing promoting improvements in gratitude and optimism. One key theoretical basis for this is the “broaden-and-build” theory which highlights pathways through which positive emotions can improve wellbeing (Fredrickson, 2001, 2013).

To our knowledge this is the first study to investigate the collective contribution of factors across three broad domains relevant to the complex construct of wellbeing. The present study is also the first empirical research to support the importance of existential positive psychology (PP2.0) involving the acceptance of suffering through tragic optimism and gratitude. Our findings therefore provide support to proposals (Fischer et al., 2020b; Holmes et al., 2020; Yamaguchi et al., 2020) that recommend the application of positive psychological approaches targeting gratitude and tragic optimism—in particular—in order to manage wellbeing during self-isolation and periods of adversity, perhaps through recently developed and innovative modules on wellbeing science that align the promotion of wellbeing with major societal challenges (Antó et al., 2021; Kemp et al., 2021). A move toward more holistic models of health that involves building wellbeing—rather than the reduction of illbeing—is necessary for promoting population wellbeing during the pandemic and beyond. Such an approach is necessary to prepare for a post-pandemic world, considering that life is often characterised by tragedy, adversity and suffering (Ivtzan et al., 2016).

## Data Availability Statement

The datasets presented in this study can be found in online repositories. The names of the repository/repositories and accession number(s) can be found at: https://osf.io/ap2rj/.

## Ethics Statement

The studies involving human participants were reviewed and approved by Department of Psychology Ethics Committee, Swansea University. The patients/participants provided their written informed consent to participate in this study.

## Author Contributions

JM and AK conceived and designed the study. JM collected and analysed the data and drafted the manuscript. JM, ZF, and AK developed the theoretical foundations as described in Mead et al. (2021). All authors provided feedback on manuscript drafts, contributed to manuscript development, and approved the submitted version.

## Conflict of Interest

JM declares that this study received funding from Fieldbay Ltd. The funder was not involved in the study design, collection, analysis, interpretation of data, the writing of this article or the decision to submit it for publication. The remaining authors declare that the research was conducted in the absence of any commercial or financial relationships that could be construed as a potential conflict of interest.
