# Supplementary material for: Protectors of Wellbeing During the COVID-19 Pandemic: Key Roles for Gratitude and Tragic Optimism in a UK-Based Cohort
Source: Front Psychol. 2021 Jul 8;12:647951. doi: 10.3389/fpsyg.2021.647951 (PMC8295471; doi:10.3389/fpsyg.2021.647951)
Supplement: Supplementary file 1 [file Data_Sheet_1.pdf]

Supplementary Material

Results – Assumption Checks

There was a significant linear relationship between the outcome variable (wellbeing) and each predictor variable, findings indicating that the assumption of linearity had been met. The histogram and p-p plot of standardised residuals indicated that the outcome variable (wellbeing) was normally distributed and the scatterplot of residuals highlighted that the data was homoscedastic. An analysis of standardised residuals was carried out on the data, showing that the data contained no outliers (SD. Residual Min = -2.17, SD. Residual Max = 2.24). Tests to assess the assumption of collinearity indicated that multicollinearity was not a concern, with the highest VIF value being 2.10 and the lowest Tolerance value being .48. The data met the assumption of independent errors (Durbin-Watson value = 2.43) and the assumption of non-zero variances.

Reliability of the Nature Connection Measure and Tragic Optimism Measure

A reliability analysis was carried out on both the Life Acceptance Measure and the Nature Connection measure as these have not been used in research previously. The Cronbach's alpha of the Life Acceptance Measure showed high internal reliability, $a = .82$ ($N = 134$), with all but one items contributing positively towards the reliability. The Cronbach's alpha highlighted the Nature Connection measure to have acceptable internal consistency, $a = .72$ ($N = 134$). Three of the four items correlated sufficiently with each other, in the range of .51 to .57. One item in the measure correlated poorly with the others, in the range of .17 to .25. Removal of this item would also increase the Cronbach's alpha score of the measure to .78. For this reason, the statement "I wish I could spend more time in nature" was removed as part of the Nature Connection measure for the regression below.
